# Breakage of a Third Generation Gamma Nail: A Case Report and Review of the Literature

**DOI:** 10.1155/2013/172352

**Published:** 2013-05-20

**Authors:** Takashi Iwakura, Takahiro Niikura, Sang Yang Lee, Yoshitada Sakai, Kotaro Nishida, Ryosuke Kuroda, Masahiro Kurosaka

**Affiliations:** Department of Orthopaedic Surgery, Kobe University Graduate School of Medicine, 7-5-1 Kusunoki-cho, Chuo-ku, Kobe 650-0017, Japan

## Abstract

The use of intramedullary nails to treat trochanteric fractures of the femur has increased with the increasing size of the elderly population. The third generation Gamma nail is currently one of the most popular devices for the treatment of trochanteric fractures. Nail breakage is a rare complication, possibly resulting from fatigue fracture of the implant. We present the first reported case of breakage of a third generation Gamma nail that was not used to treat a pathological fracture. An 83-year-old woman with an unstable trochanteric fracture of the femur was treated using a third generation Gamma nail. She was referred to our hospital 14 months postoperatively with nail breakage at the opening for the lag screw. The breakage was secondary to nonunion, which was thought to be mainly due to insufficient reduction of the fracture. The broken nail was removed, and the patient underwent cemented bipolar hemiarthroplasty. At followup 18 months later, she was mobile with a walker and asymptomatic with no complications. This case shows that inadequate operation such as insufficient reduction of the trochanteric fracture may result in nonunion and implant breakage, even when using a high-strength, well-designed implant.

## 1. Introduction

Trochanteric fractures of the femur are common in elderly individuals with osteoporosis and are usually treated surgically to facilitate early rehabilitation [1, 2]. Many devices have been developed to fix these fractures, with the most widely used being the sliding hip screw (SHS) and the intramedullary nail. In terms of load shearing, the intramedullary nail has a biomechanical advantage compared with the SHS because of its shorter lever arm [3, 4]. The use of intramedullary nails is increasing, and they are now the most commonly used fixation devices, especially for the treatment of unstable trochanteric fractures [5, 6].

The Gamma nail was introduced in the late 1980s and was the first widely available intramedullary device used for the fixation of trochanteric fractures, especially for unstable trochanteric and subtrochanteric fractures [7]. The implant consists of a sliding lag screw that passes through a short intramedullary nail and two distal locking screws that pass through the nail tip to secure it to the femoral shaft. The theoretical advantages of this device over the SHS include its minimally invasive implantation method with reduced damage to the soft tissues, a lower likelihood of infection, a possibility of shorter operative time, and its mechanical superiority [8, 9]. Excellent results have been reported with the use of this device [10–13]. However, a variety of complications have been reported. An increased incidence of secondary femoral shaft fractures was reported with use of the first generation Gamma nail compared with the SHS. These fractures were attributed largely to the first generation design features and led to modifications including downsizing of the nail [8, 14, 15]. The second generation Gamma nail was introduced in 1997 and featured decreased valgus offset, nail diameter, and number of distal locking holes, as well as a shorter length. The third generation Gamma nail was introduced in 2003 and features decreased proximal nail diameter, lag screw diameter with a new screw thread design, and distal locking screw diameter.

Although these modifications have decreased the incidence of complications, the Gamma nail is still associated with complications such as cut-out of the lag screw and nonunion and implant breakage [8, 15, 16]. Implant breakage is rare, and to our knowledge, only 2 previous cases of breakage of third generation Gamma nails have been reported, both of which were used to treat pathological trochanteric fractures [15].

We present a rare case of breakage of third generation Gamma nail due to insufficient reduction of an unstable trochanteric fracture. We also review the literature and discuss the incidence, the causes, and treatment of implant failure.

## 2. Case Report

**Figure 1:**
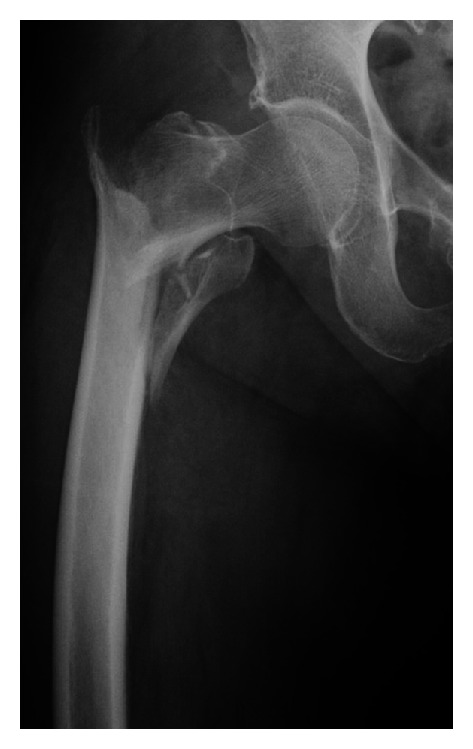
Radiograph showing an unstable trochanteric fracture of the right femur classified as 31-A2.2 according to the Orthopaedic Trauma Association classification.

**Figure 2:**
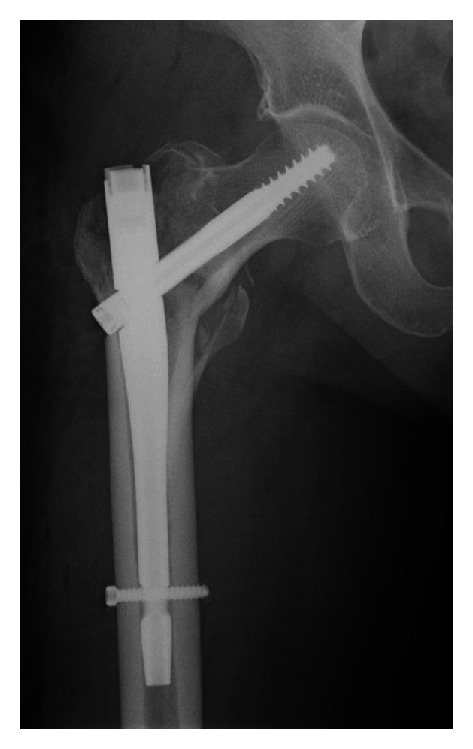
Radiograph showing insufficient reduction of the trochanteric fracture after implantation of the Gamma 3 nail.

An 83-year-old woman initially presented at another hospital with an unstable trochanteric fracture (Orthopaedic Trauma Association classification 31-A2.2) of her right femur after falling from a standing height (Figure 1). She was obese with a height of 148 cm, weight of 56 kg, and body mass index of 25.6 kg/m^2^. She had a history of hypertension, hyperlipidemia, diabetes, and cardiac arrhythmia. She underwent surgical treatment using a short Gamma 3 nail (Stryker, Tokyo, Japan) with a cervical-diaphyseal angle of 125°, a distal diameter of 10 mm, a U-lag screw, and a distal static screw. Postoperative radiography showed insufficient reduction of the fracture, with varus position of femoral head (Figure 2). Full weight-bearing with a walker was allowed immediately after surgery, and she regained mobility with a walker. 

**Figure 3:**
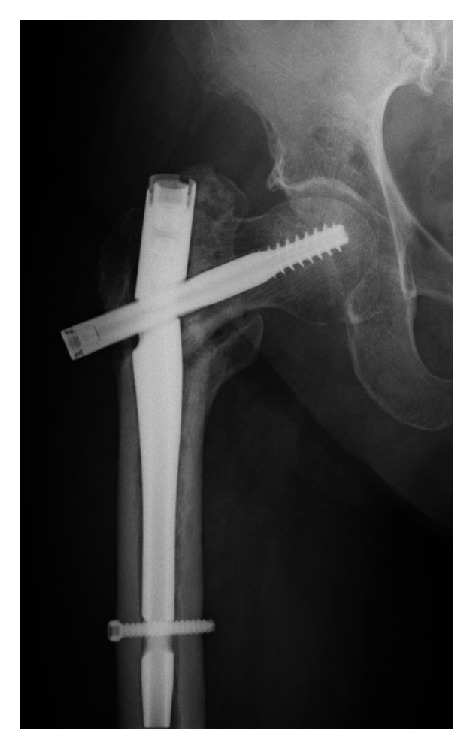
Radiograph showing nail breakage at the opening for the lag screw at 14 months after surgery. The fracture shows signs of nonunion with sclerosis of the bone ends.

At 14 months after surgery, she was referred to our institution after feeling sudden pain in her thigh without any fall or trauma and being unable to stand. Radiographs revealed breakage of the nail at the opening for the lag screw, resulting in varus angulation between the nail and the lag screw (Figure 3). The fracture showed signs of nonunion with sclerosis of the bone ends.

**Figure 4:**
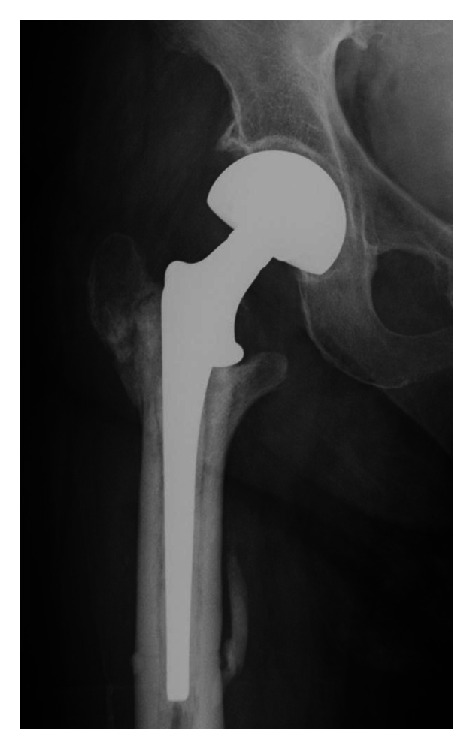
Revision surgery with cemented bipolar hemiarthroplasty.

**Figure 5:**
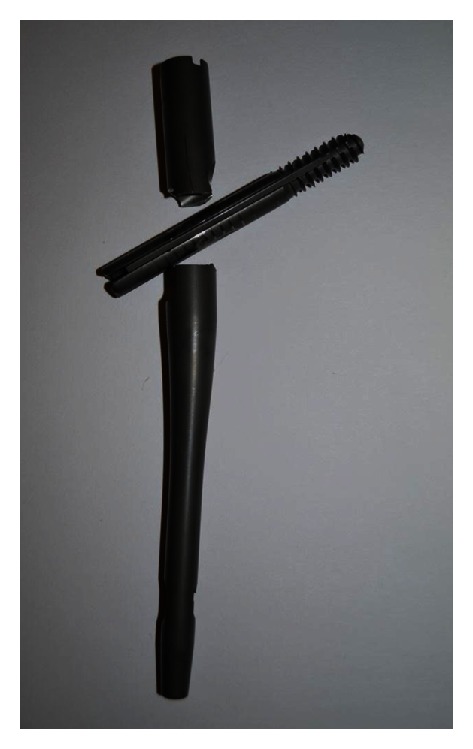
The retrieved Gamma nail, showing a horizontal fracture line at the opening for the lag screw.

The broken nail was removed, and cemented bipolar hemiarthroplasty was performed (Figure 4). The retrieved Gamma nail had a horizontal fracture line, with no obvious damage due to drilling or screw insertion (Figure 5). At 18 months after her second surgery, radiographs showed good implant alignment with no evidence of loosening. The patient was mobile with a walker and asymptomatic with no complications.

## 3. Discussion

The Gamma nail is one of the most commonly used devices for the treatment of trochanteric fractures of the femur, especially unstable fractures [6, 18]. Because of the material strength and mechanical advantage, implant failure of the Gamma nail is rare [23, 24]. We present a case of breakage of a third generation Gamma nail used to treat an unstable trochanteric fracture, which was thought to be mainly due to insufficient reduction of the fracture.

The most common cause of nail breakage is metal fatigue secondary to delayed union or nonunion [3]. Although intramedullary nails such as the Gamma nail are appropriate devices for the treatment of unstable trochanteric fractures, they are temporary implants with a limited life expectancy under continuous dynamic stress loads. In cases of delayed union or nonunion, metal fatigue caused by excessive dynamic stress can be expected [18]. Sufficient reduction to ensure stability is, therefore, necessary for unstable fractures. In the current case, the main cause of breakage of the Gamma nail was nonunion of the fracture due to insufficient reduction with varus position of the femoral head, so that the entry point of the nail was not at the tip of the greater trochanter, but at the fracture site lateral to the tip. The nonunion resulted in metal fatigue due to the continuous excessive load and eventual nail breakage. Other possible causes of breakage are the shortening of the end of the lag screw outside the lateral femur resulting in a longer lever arm and early postoperative full weight-bearing. The patient's overweight and diabetes may also have contributed to the nonunion. Regardless of the other factors implicated, surgeons should be aware that accurate reduction and fixation are important to avoid nonunion and nail breakage.

The reported incidence of breakage of Gamma nails in meta-analysis, including long Gamma nails, ranges from 0.2% to 5.7% (Table 1) [13, 17–19, 21–23, 25]. To the best of our knowledge, 40 cases of Gamma nail breakage have been reported in the literature, including 20 first generation Gamma nails, 2 second generation Gamma nails, 2 third generation Gamma nails, 14 long Gamma nails, and 2 cases with unknown nail type [3, 13, 15, 17–31]. The reported incidence of breakage of first generation Gamma nails ranges from 0.2% to 0.4% [13, 17, 18], and that of long Gamma nail ranges from 1.0% to 5.7% [19, 20, 23]. The incidences of breakage in second and third generation Gamma nails have not been reported. Two cases of breakage of third generation Gamma nails have previously been reported; both cases were in patients with a pathological fracture, which is a known risk factor for nonunion and implant failures [15]. We present the first case of breakage of a third generation Gamma nails that was not used to treat a pathological fracture.

Nails may break at different sites. Among the 40 reported cases of Gamma nail breakage, breakage occurred at the opening for the lag screw in 22 cases, at the distal locking screw in 3 cases, and along the nail shaft in 4 cases; the time of breakage ranged from 3 months to 2 years after implantation [3, 13, 15, 17–31]. The site of breakage was not described in 11 cases. All cases of breakage along the nail shaft occurred in long Gamma nails. The opening for the lag screw seems to be the weakest point, as it has a relatively small cross-sectional diameter [26]. This is the critical zone where forces from the femoral neck are transmitted to the nail in the diaphysis [22, 27]. It has been reported that inappropriate drilling of the nail at this site due to an improperly placed guide, or off-center introduction of the lag screw, may damage the nail and contribute to nail breakage [15]. Although the diameter was reduced in the third generation Gamma nail, the strength was shown to be comparable to that of second generation Gamma nail. In the current case, breakage occurred at this weak point at 14 months after surgery, with no obvious damage due to drilling or screw insertion. These findings suggest that the breakage resulted from fatigue fracture of the nail due to nonunion of the trochanteric fracture.

Salvage of failed trochanteric fracture fixation is achieved by internal fixation or arthroplasty [32–35]. The choice of salvage procedure should consider several factors including the anatomical site of the nonunion, the quality of the remaining bone and articular cartilage, and patient factors such as age and activity level. In younger patients with a well-preserved hip joint, treatment typically involves revision internal fixation with or without osteotomy or bone grafting. In older patients, however, arthroplasty is indicated to help restore function and relieve pain when there is poor bone stock or a badly damaged hip joint [35], although arthroplasty usually requires management of the discontinuous greater trochanter. Other factors such as broken hardware, deformity, and femoral bone defects also need to be considered. In our patient, we performed cemented bipolar hemiarthroplasty because of the need for the removal of the broken implant and insufficient bone stock of the femoral head. This procedure allows earlier mobilization in older patients compared with revision internal fixation [35].

In summary, we report a rare case of nail breakage in third generation Gamma nail that was treated by bipolar hemiarthroplasty. This case shows that inadequate operation such as insufficient fracture reduction may result in nonunion and implant breakage, even when using a high-strength, well-designed implant.

## Figures and Tables

**Table 1:** Meta-analyses of Gamma nail breakage.

Author	Total cases	Cases of broken nails	Nail type	Breakage site	Time	Cause of breakage
Valverde et al. [17]	223	1 (0.4%)	1st GN	Proximal	N/A	N/A

Boriani et al. [13]	1181	5 (0.4%)	1st GN	N/A	N/A	N/A

Gaebler et al. [18]	839	2 (0.2%)	1st GN	Distal	4 months	Direct trauma
			1st GN	Distal	5 months	Nonunion

Pervez and Parker [19]	35	2 (5.7%)	Long GN	Middle	3 months	Delayed union
			Long GN	N/A	5 months	Delayed union (PF)

Van Doorn and Stapert [20]	101	2 (2.0%)	Long GN	Proximal	7 months	Nonunion (PF)
			Long GN	Middle	9 months	Nonunion (PF)

Docquier et al. [21]	439	1 (0.2%)	1st or 2nd GN	N/A	N/A	Delayed union

Álvarez et al. [22]	843	5 (0.6%)	1st GN	Proximal	7 months	Nonunion
			1st GN	Distal	7 months	Nonunion
			2nd GN	Proximal	7 months	Nonunion
			Long GN	Middle	10 months	Nonunion
			Long GN	Proximal	8 months	Nonunion

Sehat et al. [23]	100	1 (1.0%)	Long GN	Middle	N/A	Insufficient reduction

1st GN: the first generation Gamma nail, 2nd GN: the second generation Gamma nail, Long GN: long Gamma nail, Proximal: the opening for the lag screw, middle: nail midshaft, distal: the opening for the distal locking screw, N/A: not available in the literature, and PF: pathological fracture.
